# Reversibilidade da Cardiomiopatia Dilatada na Síndrome de Sheehan: Um Relato de Caso

**DOI:** 10.36660/abc.20190547

**Published:** 2021-02-02

**Authors:** Maria Luíza Barbosa Fernandes Dourado, Taís Paiva da Costa, Márcia Sampaio de Carvalho, Carlos Geraldo Guerreiro de Moura

**Affiliations:** 1 Obras Sociais Irmã Dulce SalvadorBA Brasil Obras Sociais Irmã Dulce, Salvador, BA - Brasil; 2 Escola Bahiana de Medicina e Saúde Pública SalvadorBA Brasil Escola Bahiana de Medicina e Saúde Pública, Salvador, BA - Brasil

**Keywords:** Hipopituitarismo, Síndrome de Sheehan, Cardiomiopatia Dilatada, Diagnóstico por Imagem, Terapia de Reposição Hormonal

## Introdução

A síndrome de Sheehan, descoberta em 1937 por Harold Leeming Sheehan, é descrita como pan-hipopituitarismo secundário à necrose hipofisária após hemorragia pós-parto.^1^ A apresentação do quadro clínico depende da deficiência hormonal apresentada, e pode envolver alterações nos níveis de cortisol sérico, função tireoidiana, hormônios do crescimento, amamentação e hormônios sexuais.^2^ Embora escassamente descrita na literatura, há relatos de miocardiopatia dilatada associada à síndrome de Sheehan, alguns com reversibilidade da cardiopatia após terapia de reposição hormonal.^3^ Este trabalho relata um caso clínico de síndrome de Sheehan associada à miocardiopatia dilatada que apresentou melhora da função cardíaca após terapia de reposição hormonal.

## Relato de Caso

O presente artigo é sobre uma mulher de 36 anos, casada, do lar, natural de Inhambupe/BA, que havia sido hospitalizada em serviço médico terciário com dispneia progressiva há 2 meses que evoluiu para dispneia em repouso 02 dias após a hospitalização. Além disso, ela relatou edema de membros inferiores e edema periorbital. Também se queixava de náuseas e vômitos pós-prandiais por 01 semana com restos de comida, sem muco ou sangue, afebril. Referia internação anterior, aos 18 anos, devido a complicações decorrentes de pré-eclâmpsia e hemorragia pós-parto, negou transfusões de sangue. Também descreveu agalactia e amenorreia pós-parto. A paciente tem uma vida sexual ativa com um único parceiro e não utiliza nenhum método contraceptivo. Ao exame físico, estado geral regular, com fala confusa, hipoatividade e hipotensão (∆PAS 100-80 mmHg x ∆PAD 70-50 mmHg). Pele com turgor e elasticidade reduzidos, presença de edema periorbital. O sistema cardiovascular apresentava precórdio calmo, impulso apical não palpável e não visível, sons cardíacos hipofonéticos, sem sopros, sem som cardíaco extra. Extremidades com perfusão, com edema depressível + 1/4 +, frio, indolor. Outros acompanhamentos sem alterações.

Os exames laboratoriais iniciais mostraram TSH inadequadamente normal em 4,93 µUI/mL (0,38-5,3) com T4 livre abaixo de 0,4 ng/dL (0,5-1,2), hiponatremia normovolêmica (sódio 133mEq/ L - VR 136-144). Exames complementares na hospitalização: hemoglobina 12 mg/dL, hematócrito 35,9%, leucograma 12.880: 89% segmentados, 4% linfócitos, 1% eosinófilos e 6% monócitos; plaquetas 165.000 / mm³ e função renal normal.

A reposição de hidrocortisona em bolus de 500 mg foi seguida de levotiroxina em baixas doses (50 mcg / dia). Após introdução da terapia hormonal, a paciente apresentou melhora da hipoatividade e astenia apresentada na hospitalização.

**Figura 1:**
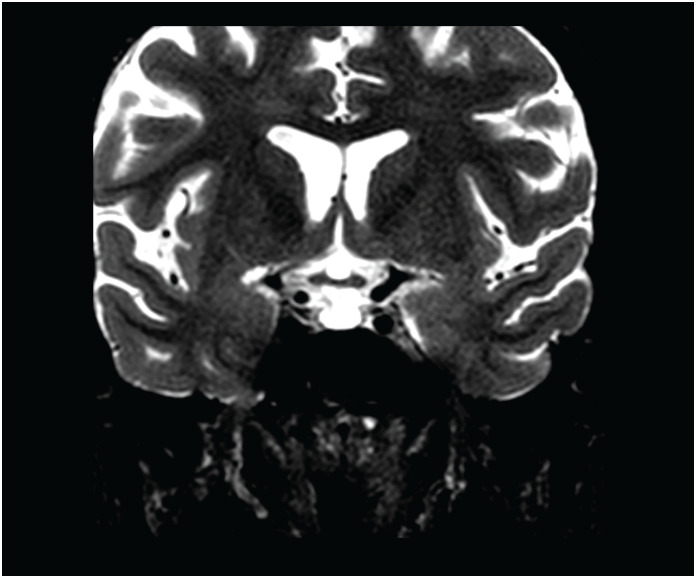
Ressonância magnética da sela túrcica parcialmente vazia.

O quadro clínico e a resposta à hormonioterapia confirmaram a suspeita diagnóstica de hipopituitarismo secundário à necrose hipofisária após hemorragia pós-parto, confirmado pelos seguintes testes: GH 0,1 ng/mL (0,5-3,6), beta-estradiol 20 pg/mL (<40 pós-menopausa), FSH 4,5 mUI/mL (16 – 113: pós-menopausa), LH 2,96 mIU/mL (10,8 – 58,6: pós-menopausa), prolactina 3,36 ng/mL (2-15), ACTH 35,8 pg/mL (VR 7,2-63,3) e cortisol sérico matinal 1,5 mcg/dL (5,4-25). A ressonância nuclear magnética cerebral revelou sela túrcica vazia parcial, com herniação da cisterna supra-selar para o interior da sela túrcica, identificando uma fina camada de glândula pituitária no assoalho selar, com realce homogêneo ao meio de contraste (Figura 1).

**Figura 2:**
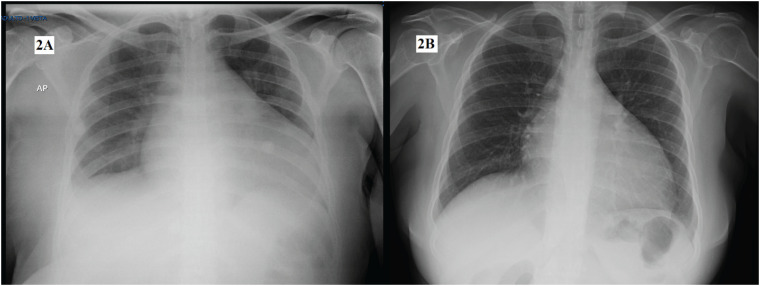
A) Radiografia de tórax na hospitalização. B) Radiografia de tórax após terapia hormonal.

Ciente do quadro de dispneia e hipofonia dos sons cardíacos associado à síndrome edemigênica, foram solicitadas radiografia de tórax (Figura 2A) e ecocardiografia transtorácica. A radiografia mostrou a presença de cardiomegalia. O ecocardiograma mostrou miocardiopatia dilatada com disfunção sistólica ventricular esquerda significativa, às custas de hipocinesia difusa, fração de ejeção do ventrículo esquerdo (FEVE) de 27% e regurgitação mitral leve.

Uma vez realizados os ajustes da hormonioterapia instituída com levotiroxina 100 mcg/dia e prednisona 10 mg/dia, houve melhora clínica e radiológica significativa (Figura 2B). O ecocardiograma serial após 2 semanas de tratamento mostrou melhora de 12% na fração de ejeção e redução da disfunção sistólica global, mesmo sem terapia específica para Insuficiência Cardíaca Congestiva (ICC).

A terapia para insuficiência cardíaca com redução da fração de ejeção só foi introduzida uma semana antes da alta hospitalar, pois até o momento a paciente apresentava níveis pressóricos limítrofes. Ela foi encaminhada ao ambulatório de cardiologia, com orientações sobre o uso de betabloqueadores cardiosseletivos, além de espironolactona.

## Discussão

A incidência da síndrome de Sheehan, secundária à hemorragia periparto, está diretamente relacionada à qualidade do atendimento médico durante a gravidez.^4^ A mortalidade materna é um importante marcador do estado de saúde da população. Uma das principais causas de mortalidade materna é a hemorragia pós-parto, que pode ter como consequência a ocorrência de necrose hipofisária.^5^

A apresentação clínica da síndrome de Sheehan depende do nível de isquemia da glândula pituitária.^1^ Cerca de 75% das células da hipófise precisam estar comprometidas para causar deficiência hormonal secundária.^3^ Os sinais e sintomas são divididos em doença aguda e crônica.^6^ Os sintomas agudos consistem em hipotensão, choque, taquicardia, hipoglicemia, hiponatremia, fadiga extrema, náuseas e vômitos, classicamente representados por insuficiência adrenal aguda. Cronicamente, os pacientes podem apresentar astenia, fadiga, diminuição da força muscular, constipação, intolerância ao frio relacionada ao hipotireoidismo central; redução da libido, agalactia, amenorreia e infertilidade, devido à redução do estímulo gonadotrófico; incluindo transtornos psiquiátricos.^1^

Segundo a literatura, a busca por atendimento médico é motivada por distúrbios hidroeletrolíticos, principalmente a hiponatremia. Durante os primeiros atendimentos à paciente do caso, náuseas, vômitos e hiponatremia direcionaram a abordagem diagnóstica e terapêutica. Só foi possível saber a história obstétrica da paciente após a resolução do estado confusional. Porém, no contexto de emergência, a paciente foi internada com síndrome edemigênica de etiologia cardíaca.

A apresentação da Síndrome de Sheehan como ICC é atípica e o envolvimento cardíaco foi considerado o mais raro entre os descritos.^7^ Em 2013, Doshi et al.,^3^ já haviam mencionado a entidade hipo-poliglandular associada à reversibilidade da função cardíaca. A cardiopatia do paciente com hipotireoidismo está associada principalmente ao derrame pericárdico, quando o tempo de enchimento ventricular é reduzido, podendo resultar em tamponamento cardíaco.^8^ Quando relacionada à insuficiência adrenal, é relatada em pacientes com hipocortisolismo como parte das síndromes poliglandulares autoimunes do tipo 1, também reversíveis após correção hormonal.^9^ No entanto, a etiologia da miocardiopatia dilatada relacionada à síndrome de Sheehan permanece desconhecida.

A melhora da função ventricular demonstrada no quadro clínico durante o curto período de duas semanas foi peculiar. Houve um aumento da fração de ejeção de 27% para 39% após duas semanas de hospitalização, apesar do uso de terapia formal para insuficiência cardíaca. Outros casos descritos na literatura expõem a reversibilidade da miocardiopatia dilatada quando associada à síndrome de Sheehan; porém, a maioria deles associando reposição hormonal e terapia direcionada à ICC com fração de ejeção reduzida.^7^^,^^9^^–^^12^

Doshi et al.,^3^ abordaram o caso clínico de uma paciente de 42 anos com uma apresentação de emergência de choque cardiogênico secundário a pan-hipopituitarismo devido à síndrome de Sheehan. Amenorreica há 14 anos (data da última gestação), a paciente foi tratada com glicocorticoide, levotiroxina e 48 horas de uso de inotrópico. Seis meses após o início da terapia, a paciente apresentou aumento de 100% da FEVE (inicial: 20%, seguimento: 40%), melhora dos parâmetros radiológicos e tornou-se assintomática. Em 2014, na Arábia Saudita, foi estudado o caso de uma jovem paciente que deu entrada no pronto-socorro apresentando dispneia e com síndrome edemigênica há 6 meses com diagnóstico de miocardiopatia dilatada periparto. Porém, após extensa investigação, o diagnóstico inicial foi reconsiderado, pois a paciente apresentava história de hemorragia periparto, insuficiência adrenal e tireoidiana, além de sela túrcica vazia. Assim, ela foi diagnosticada com síndrome de Sheehan associada à miocardiopatia dilatada, revertida em 06 meses após a reposição hormonal.^7^

Concluiu-se que a síndrome de Sheehan associada à miocardiopatia dilatada é rara e não há abordagem terapêutica descrita na literatura. A reposição hormonal para as deficiências apresentadas é o principal recurso disponível conhecido, uma vez que a melhora dos casos clínicos descritos independe da terapia específica para ICC com fração de ejeção reduzida. As principais síndromes tratadas envolvem a reposição do hormônio tireoidiano e corticoterapia, não havendo consenso sobre o benefício da reposição de GH.^1^
