# Supplementary material for: Reversibilidade da Cardiomiopatia Dilatada na Síndrome de Sheehan: Um Relato de Caso
Source: Arq Bras Cardiol. 2021 Feb 2;116(2 Suppl 1):17–20. [Article in Portuguese] doi: 10.36660/abc.20190547 (PMC8118631; doi:10.36660/abc.20190547)
Supplement: Translation

# Translation: Dilated Cardiomyopathy Reversibility in Sheehan's Syndrome: A Case Report

**Authors:** Maria Luíza Barbosa Fernandes Dourado, Taís Paiva da Costa, Márcia Sampaio de Carvalho, Carlos Geraldo Guerreiro de Moura

**Affiliations:** 1 SalvadorBA Brazil Obras Sociais Irmã Dulce, Salvador, BA - Brazil; 2 SalvadorBA Brazil Escola Bahiana de Medicina e Saúde Pública, Salvador, BA - Brazil

**Keywords:** Hypopituitaris, Sheehan's Syndrome, Cardiomyopathy, Dilated, Diagnostic, Imaging, Hormone Replacement Therapy

## Introduction

Sheehan's syndrome, discovered in 1937 by Harold Leeming Sheehan is described as pan-hypopituitarism secondary to pituitary necrosis after post-partum hemorrhage.^1^ The clinical condition presentation depends on the hormonal shortfall presented, and may involve changes in serum cortisol levels, thyroid function, growth hormones, breastfeeding and sex hormones.^2^ Although poorly described in the literature, there are reports of dilated cardiomyopathy associated with Sheehan's syndrome, some with reversibility of cardiopathy after hormone replacement therapy. ^3^ This paper reports a clinical case of Sheehan's syndrome associated to dilated cardiomyopathy that displayed cardiac function improvement after hormone replacement therapy.

## Case Report

The subject reported on this paper is about a 36-year-old female, married, housekeeper, from Inhambupe/BA, which had been admitted to a tertiary medical service with progressive dyspnea for 2 months that evolved to resting dyspnea at 02 days from admission. In addition, she reported lower limb edema and periorbital edema. Also complained about postprandial nausea and vomiting for 01 week with food remains, without mucus or blood, afebrile. Also referred asthenia, somnolence and mental confusion, with difficulty in the chronological organization of the facts. Thus, the patient had been treated with diuretic therapy in emergency, with partial improvement of dyspnea and pulmonary edema. She also reported previous hospitalization at age 18 due to complications caused by preeclampsia and post-partum hemorrhage, having no blood transfusions. She claimed post-partum agalactia and amenorrhea 18 years ago. The patient has an active sexual life with a single partner and doesn't use any contraceptive method. At physical examination, regular general state, with confused speech, hypoactivity and hypotension (∆PAS 100-80 mmHg x ∆PAD 70-50 mmHg). Skin with reduced turgor and elasticity, presence of periorbital edema. The cardiovascular system displayed calm precordium, non-palpable and non-visible apical impulse, hypophonetic heart sounds, no blows, no extra heart sound. Perfused extremities with depressible edema + 1/4 +, cold, painless. Other follow-ups without changes.

Initial laboratory tests showed inadequately normal TSH in 4.93 µUI/mL (0.38-5.3) with free T4 under 0.4 ng/dL (0.5-1.2), normovolemic hyponatremia (sodium 133mEq/L - VR 136-144). Additional admission exams: hemoglobin 12 mg/dL, hematocrit 35.9%, leukogram 12,880: 89% segmented, 4% lymphocytes, 1% eosinophils and 6% monocytes; platelets 165,000/mm³ and normal renal function.

The replacement of hydrocortisone 500 mg bolus was followed by low doses of levothyroxine (50 mcg/day). After introduction of hormonal therapy, the patient presented improvement of hypoactivity and asthenia presented on admission.

The clinical status and response to hormone therapy confirmed the diagnostic suspicion of hypopituitarism secondary to pituitary necrosis after post-partum hemorrhage; confirmed by the following tests: GH 0.1 ng/mL (0.5-3.6), beta-estradiol 20 pg/mL (<40 post-menopausal), FSH 4.5 mUI/mL (16 – 113: post-menopausal), LH 2.96 mIU/mL (10.8 – 58.6: postmenopausal), prolactin 3.36 ng/mL (2-15), ACTH 35.8 pg/mL (VR 7.2-63.3) and morning serum cortisol 1.5 mcg/dL (5.4-25). Brain's magnetic nuclear resonance revealed partial empty *sella turcica* with herniation of the suppressing cistern into the saddle, identifying a thin layer of the pituitary gland in the sealing floor, with homogeneous enhancement to the medium contrast (Figure 1).

**Figure 1:** Magnetic resonance of the partial empty sella turcica.

Aware of dyspnea and cardiac sounds hypophonese status associated with edemigenic syndrome, a chest X-ray (Figure 2.A) and transthoracic echocardiography were requested. The radiograph pointed to cardiomegaly. The echocardiogram displayed dilated cardiomyopathy with significant left ventricular systolic dysfunction, at the expense of diffuse hypokinesia ventricular ejection fraction (LVEF) of 27%, and mild mitral regurgitation.

**Figure 2:** A) Chest radiography of hospital admission. B) Chest radiography after hormone therapy.

Once adjustments of the hormone therapy instituted were accomplished with levothyroxine 100 mcg/day and prednisone 10 mg/day, significant clinical and radiological (Figure 2B) improvement had been revealed. The serial echocardiogram after 2 weeks of treatment showed a 12% improvement in ejection fraction and reduction of global systolic dysfunction even without specific therapy for congestive heart failure (CHF).

Heart failure therapy with reduced ejection fraction was only introduced a week before hospital discharge, as to date, the patient had borderline blood pressure levels. She was referred to the cardiology outpatient clinic with guidance on the use of cardioselective beta-blockers, in addition to spironolactone.

## Discussion

Sheehan's syndrome incidence, secondary to peripartum hemorrhage, is directly related to quality of medical care during pregnancy.^4^ Maternal mortality is an important marker of population's health status. One of the main causes of maternal mortality is post-partum hemorrhage with may have as a consequence the occurrence of pituitary necrosis.^5^

The clinical presentation of Sheehan's syndrome depends on the level of ischemia of pituitary's gland.^1^ About 75% of pituitary's cells should be compromised to cause secondary hormone deficiency.^3^ Signs and symptoms are divided into acute and chronic illnesses.^6^ The acute ones consisted of hypotension, shock, tachycardia, hypoglycemia, hyponatremia, extreme fatigue, nausea and vomiting, classically represented by acute adrenal insufficiency. Chronically, patients may present asthenia, fatigue, muscle strength decrease, constipation, cold intolerance related to central hypothyroidism; reduction of libido, agalactia, amenorrhea and infertility, due to gonadotrophic stimulus reduction; including psychiatric disorders.^1^

According to literature, the search for medical care is motivated by hydroelectrolytic disturbances, especially hyponatremia. During the first care given to the patient of the case, nausea, vomiting and hyponatremia directed the diagnostic and therapeutic approach. It was only possible to know patient's obstetric history after confusional status was solved. However, in the emergency context, the patient was admitted with edemigenic syndrome of cardiac etiology.

The presentation of Sheehan's Syndrome as CHF is atypical and cardiac involvement was considered the rarest among those described.^7^ By 2013, Doshi et al.^3^ had already mentioned the hypo-polyglandular entity associated to cardiac function reversibility. The cardiopathy of the patient with hypothyroidism is mainly associated with the pericardial effusion, when the ventricular filling time is reduced, sometimes resulting in cardiac tamponade.^8^ When related to adrenal insufficiency, it is reported to patients with hypocortisolism as part of type 1 autoimmune polyglandular syndromes, also reversible after hormonal correction.^9^ However, etiology of the dilated cardiomyopathy related to Sheehan's syndrome remains unknown.

The improvement of ventricular function demonstrated in the clinical status during the short period of two weeks was curious. There was an ejection fraction increase from 27% to 39% after two weeks of admission despite the use of formal heart failure's therapy. Other cases described in the literature expose the reversibility of dilated cardiomyopathy when associated with Sheehan's syndrome, however, most of them associating hormone replacement and CHF with LVEF's therapy.^7^^,^^9^^–^^12^

Doshi et al.^3^ approached the clinical case of a 42-year-old female patient with an emergency presentation of secondary cardiogenic shock to pan-hypopituitarism due to Sheehan's syndrome. Amenorrheic 14 years ago (date of the last gestation), she was managed with glucocorticoids, levothyroxine and 48 hours of inotropic use. Six months after the therapy started, the patient had a 100% increase of LVEF (initial: 20%, follow-up: 40%), radiological parameters improvement and became asymptomatic. In 2014, in Saudi Arabia, the case of a young patient who was admitted to the emergency room presenting dyspnea and edemigenic syndrome for 6 months with a peripartum dilated cardiomyopathy diagnosis was studied. However, after extensive investigation, the initial diagnosis was reconsidered, since the patient had a history of peripartum hemorrhage, adrenal and thyroid insufficiency, as well as empty *sella turcica*. Thus, she was diagnosed with Sheehan's syndrome associated with dilated cardiomyopathy, reversed in 06 months after hormone replacement.^7^

It was concluded that Sheehan's syndrome associated with dilated cardiomyopathy is rare and there is no therapeutic approach described by literature. The hormone replacement for the deficiencies presented is the main known available resource, since the improvement of the clinical cases described is independent of the specific therapy for CHF with reduced ejection fraction. The main syndromes treated involve the replacement of thyroid hormone and corticoid therapy, and there is not a consensus about the benefit of GH replacement.^1^
